# Long-Term Follow-Up of Sepsis Induced Immunoparalysis

**DOI:** 10.1186/2197-425X-3-S1-A49

**Published:** 2015-10-01

**Authors:** M Raja, HDT Torrance, ER Longbottom, AJ Stroud, ME Vivian, PS Zolfaghari, RM Pearse, CJ Hinds, MJ O'Dwyer

**Affiliations:** The Royal London Hospital, Barts Health Trust, Adult Critical Care Unit, London, United Kingdom; Queen Mary University of London, William Harvey Research Institute, London, United Kingdom

## Introduction

Severe sepsis induces a state of immunoparalysis.[1] Animal models have demonstrated this to be secondary to microbial-induced host epigenetic alterations, which persist and are associated with long-term immunoparalysis.[2] Whilst human sepsis is associated with poor long-term outcomes in conjunction with recurrent infections,[3] it is not clear if the immunoparalysed state persists following recovery from the initial septic insult.

## Objectives

1. To confirm the presence of circulating mediators capable of causing immunoparalysis following bacteraemia.

2. To assess for the presence of immunosuppressive mediators following hospital discharge with full functional recovery.

## Methods

Consecutive adult patients (n = 7) with bacteraemia and an admission diagnosis of infection were recruited. Serum was collected at 3 time points; within 48 hours of the positive blood culture, 5 days later & 12 months following hospital discharge. Peripheral blood mononuclear cells (PBMCs) were collected from a healthy control cohort (n = 7) and pooled. Healthy PBMCs were co-cultured with 30% septic serum for 20 hours with and without GM-CSF (200ng/ml). CD14^+^HLA-DR (mHLA-DR) geometric-mean fluorescent intensity (MFI) was determined using flow cytometry. Data were analysed with non-parametric statistics with results presented as median & IQR .

## Results

**Figure 2:**
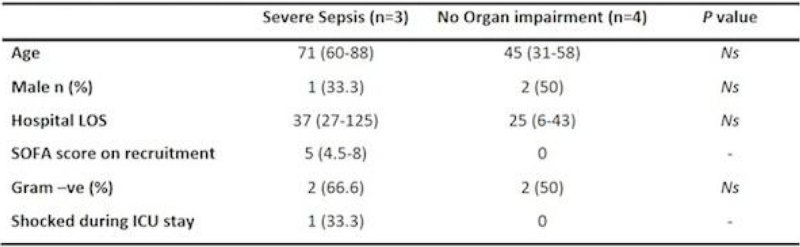
**Demographic and clinical details of patients requiring ICU care (severe sepsis) and those receiving ward based care (no organ impairment).**

Three patients required ICU care & four were managed on the ward. Demographic & clinical data are presented in Figure 2. mHLA-DR levels were lower when PBMCs were co-cultured with the baseline bacteraemic sample in comparison with control group serum (Figure 1, *P* = 0.01). mHLA-DR levels following co-culture with the baseline sample were not significantly different to the levels observed when co-cultured with day 5 serum (Figure 1A). in comparison to the baseline sample, when co-cultured with serum from 12 months, mHLA-DR levels were increased (Figure 1A, *P* = 0.007). mHLA-DR levels at 12 months were not different from those seen when control serum was used (Figure 1A*P* = 0.85). mHLA-DR levels were higher in the presence of GM-CSF when co-cultured with baseline (Figure 1B, *P* = 0.01) and 12 month (Figure 1B, *P* = 0.02) samples but not with day 5 serum (Figure 1B, *P* = 0.17).

**Figure:**
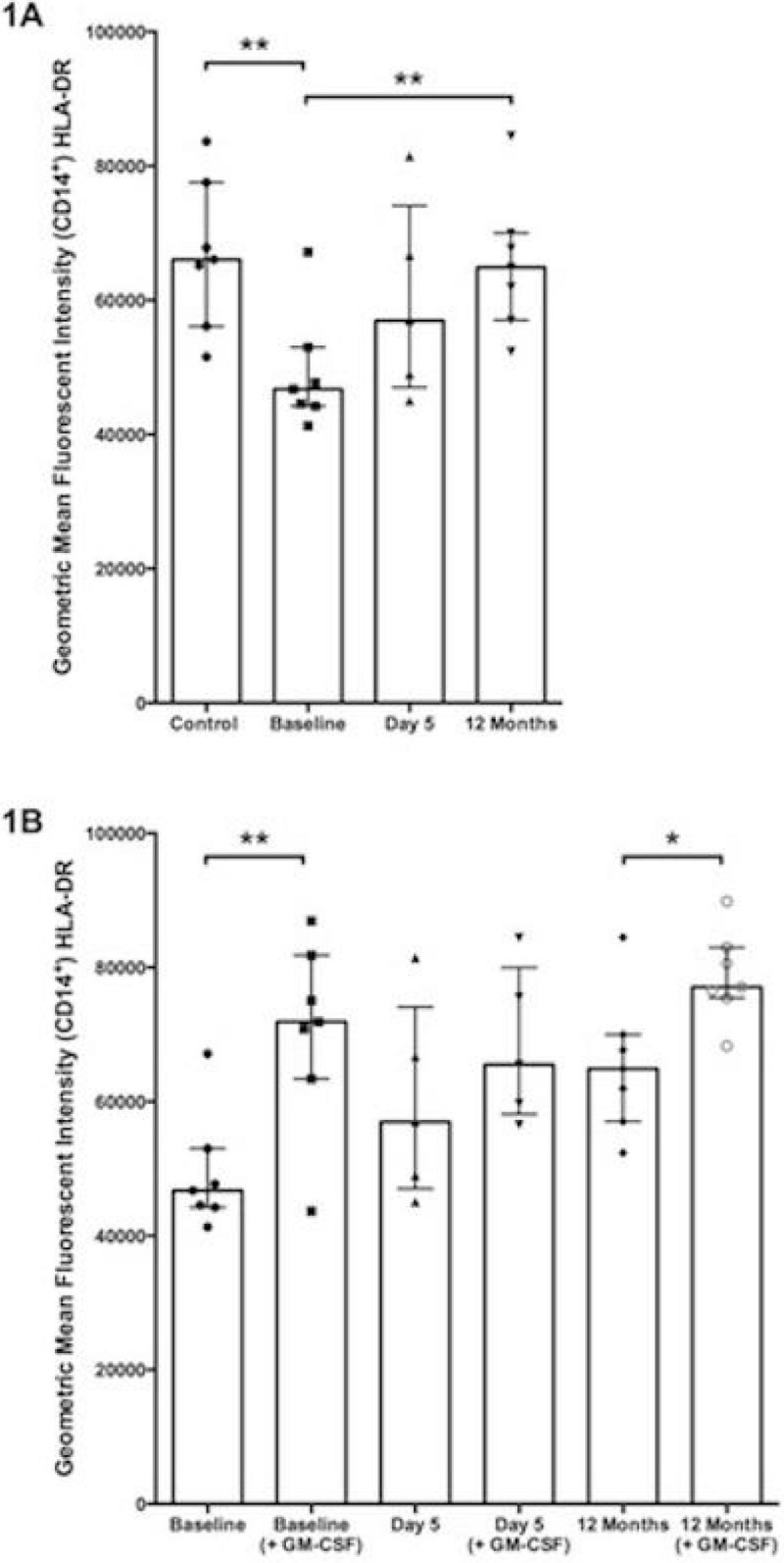
Figure 1

## Conclusions

Circulating mediators present in the serum of bacteraemic patients reduces the ability of healthy monocytes to express cell surface HLA-DR, which is reversible in the presence of an immunostimulant. Serum from patients following full recovery from the acute illness does not reduce HLA-DR expression on healthy monocytes.

## Grant Acknowledgment

ESICM Basic Science Award 2014.
